# Priapism: Comorbid Factors and Treatment Outcomes in a Contemporary Series

**DOI:** 10.1155/2012/672624

**Published:** 2012-07-03

**Authors:** Henry P. Gottsch, Richard E. Berger, Claire C. Yang

**Affiliations:** Department of Urology, University of Washington, USA

## Abstract

*Objective*. The goal of this study is to describe comorbid characteristics in patients who have priapism, and their treatment outcomes. *Methods*. Chart review was undertaken on men who had a diagnosis of priapism from a tertiary medical center, from 2000–2010. Men with priapism due exclusively to the use of prescription erectile aids and medications were not included in the review. *Results*. We identified 79 patients with the priapism. The most common type of priapism was the low flow variant. High flow priapism was identified in 2 patients. The most common general comorbid condition associated with priapism was mental illness (including substance abuse), which was present in 56% of the patients. Neurogenic priapism accounted for 19% of the total priapism events. Psychopharmaceutical agents and recreational drugs were commonly associated with ischemic priapism. Acute complications of priapism treatment were not common, but long-term complications, especially erectile dysfunction, were frequent. *Conclusions*. We describe the characteristics and outcomes of a large group of patients with priapism. Our experience at a tertiary care center indicates that mental illness, including substance abuse disorders, is a highly prevalent comorbid condition in men who experience priapism. Consistent with previous reports, erectile dysfunction is the most common complication from priapism and its treatment, occurring in the majority of men.

## 1. Introduction

Priapism is a relatively uncommon occurrence and as a result, there is scant contemporary literature on the associated medical conditions of men who experience priapism. The occurrence of priapism and its treatment have been historically reported in small case series, and this literature was comprehensively reviewed in aggregate by an expert panel of American Urological Association in 2003 and reconfirmed in 2010 [1]. There are well-recognized associations between priapism and predisposing factors such as hematologic disorders and particular medications, but better understanding of other characteristics of affected men could potentially aid in their management. The objective of this retrospective review is to report on a contemporary cohort of men presenting to a tertiary care medical center with priapism, describing comorbidities and treatment outcomes. 

## 2. Methods

The data for this study was drawn from a tertiary care center comprised of a university hospital and a level I trauma center/county hospital. The hospitals' Institutional Review Board approved this study. We searched hospital billing records from 2000–2010 for ICD-9 code (607.3) for priapism. Medical record review was undertaken on men who had a diagnosis of priapism, with particular focus on cause of priapism, concomitant comorbid conditions, interventions to treat priapism, and subsequent complications and sequelae. A clinical diagnosis of priapism was made based on a history and physical examination, with a penile blood gas performed in most cases. Comorbid conditions were determined through chart review. Patients who were seen with priapism due to physician-prescribed erectile dysfunction (ED) treatment were excluded from this cohort. Mental health disorders were included as comorbid conditions if they were previously documented in the medical record. Substance abuse was considered as a mental health disorder and was noted if there was preceding history of emergency room visits, medical treatment, positive toxicology screen (not available on all patients), or disability associated with substance abuse. Duration of use and timing of substance abuse in relation to the priapism event was not available.

Treatment generally conformed to the AUA guidelines for priapism [1]. The selection of a shunt procedure was at the discretion of the surgeon, but generally began with distal penile shunting, and then proceeded to proximal shunting, as needed. The distal corporo-glanular shunting procedures included the Al-Ghorab, Winter, Ebbehoj, and T-Shunt (with and without tunneling). Distal shunting was carried out in the both the emergency room and in the operating room settings. Proximal shunting techniques included corporo-spongiosal shunting done via transscrotal or perineal approach and one patient who had a corporal-dorsal venous shunt (Grayhack). All proximal shunts were carried out in the operating room. Recovery of erectile function was dichotomized to able to complete intercourse or not to able to complete intercourse or not. No standardized measurement of erectile function was available. Complications were reported based on the Dindo-Claviean classification [2]. 

## 3. Results

### 3.1. Diagnosis

We identified a total of 115 patients with diagnosis coding and chart review consistent with priapism of these there were 79 patients that had priapism not related to prescription ED medication treatment. Seventy-seven patients had low-flow priapism, 62 of whom had ischemic, low-flow priapism confirmed in part by a blood gas of pH < 7.25 (Table 1). Two patients presented with high-flow priapism. In those patients, Doppler ultrasound, pudendal arteriography, and embolization were used for further diagnosis and treatment.

### 3.2. Comorbid Conditions Associated with Priapism. 

Comorbid conditions are listed in Table 2. The most common comorbidity associated with ischemic priapism was mental illness, a diagnosis found in 56% of the patients. The most common form of mental illness was substance-abuse disorder (38%). Other comorbid mental health diagnoses included bipolar disorder, schizophrenia and depression. Of the 19 patients with active diagnoses of bipolar disorder, schizophrenia, or depression only 5 reported having taken their medications as prescribed. 

Use or abuse of psychopharmaceuticals (not necessarily with a preexisting diagnosis of substance use disorder) was a very common factor in the low-flow, ischemic priapism patients. Cocaine was the most commonly abused recreational drug associated with priapism (*n* = 10). Other substances included alcohol, narcotics, and amphetamines. The most common prescription medication associated with priapism was trazodone (*n* = 9), used both as a sleep aid and a substance of abuse. Other common psychopharmaceuticals were in the antipsychotic/neuroleptic class of medications (*n* = 5). It was not possible to determine if all prescription medications were being used as prescribed. 

The second most common comorbid condition was neurologic injury (*n* = 15). Each of the 15 patients identified with neurogenic priapism had suffered an acute injury of the central nervous system prior to the onset of priapism. This type of priapism accounted for 19% of the low-flow priapism events. Classified as low-flow, the nature of neurogenic priapism is distinct in that the duration of priapism was much shorter than that associated with the typical ischemic low-flow priapism. Furthermore, the erection typically resolved spontaneously. The majority of patients in this group had an injury that was associated with the cerebrum (*n* = 8), followed by both cerebral and spinal cord lesions (*n* = 4), and isolated spinal cord lesions (*n* = 3). 

Other comorbid factors in this group included hypertension, HIV, and sickle cell disease. Only 14 of the 77 patients (18%) with low-flow priapism were otherwise healthy with no psychiatric, substance abuse disorders, or systemic medical conditions. 

High-flow priapism occurred in only 2 patients, both with high-flow, arteriovenous fistulas associated with trauma. 

### 3.3. Priapism Treatment and Outcomes

 Twenty nine of the priapism patients initially presented at outside facilities. Nineteen were evaluated by a urologist prior to transfer to our facility, and 6 patients had undergone shunting prior to transfer.

Of the two patients with high-flow priapism, one underwent embolization, and the other declined treatment and was lost to followup. 

The 77 patients with low-flow priapism included 15 with an acute neurologic insult (e.g., spinal cord injury, head trauma) and were considered to be a distinct subgroup of ischemic priapism. Neurogenic priapism spontaneously resolved without treatment, typically within 6 hours of onset. The remaining 62 patients with low-flow priapism were treated initially with corporal aspiration and irrigation, followed by shunting when deemed appropriate. Thirty-six patients required at least one shunt procedure, and 18 patients required two or more separate shunts. There were 5 patients who had no interventions. 

Outcomes of priapism treatment are listed in Table 3. Immediate complications were seen in 6 patients. Atrial fibrillation requiring cardioversion was the only complication reported with corporal irrigation (Clavian grade IIIa). This was presumed to be a complication of the phenylephrine irrigation, which has not been previously reported in the literature. Of the 5 subjects who had complications following shunt procedures, 2 had urethral injuries (Clavian grade 1). Both patients required multiple shunt procedures to achieve sustained detumescence.

After an emergency department visit or hospital admission for priapism, the clinic follow-up rate for patients was 44%, with a mean followup of 8 months. Nine of 22 (41%) men who underwent penile irrigation only were seen in followup, whereas of the patients who underwent shunting 26 of 36 (72%) returned for follow-up care. Chronic genital pain, defined as a need for ongoing prescription medical therapy >6 weeks after the priapism event, was reported in 5 patients at followup; of these, 4 underwent proximal shunting. Ischemic time for the patients with pain ranged from 30 to 140 hrs, and this was not significantly different than those without chronic pain (*P* > 0.05).

 Preservation of erectile function adequate for intercourse with or without erectile aids was uniformly poor, and adequate erections were reported in only 6 of the 35 patients who had followup. All 6 patients with return of erectile function had shunts. Successful distal shunts included Winter's (*n* = 2) and Al-Ghorab (*n* = 1). Quackel's shunting or corporo-spongiosal shunting was successful in the remaining 3 patients. Age less than 45 years old and duration of priapism less than 48 hrs were the only commonality of patients with return of erectile function. None of the follow-up patients treated with irrigation only reported return of erectile function. Given the limited number of patients with followup no statically significant inference could be made regarding the duration of priapism and timing of procedural intervention on erectile function. Corporo-spongiosal shunts (Quackel type only) were surgically closed in 5 patients, with the goal of restoring erectile function. Following shunt closure, 2 patients had return of erections sufficient for intercourse. Three patients went on to penile prosthesis placement, 1 following T-shunt with tunneling and 2 following proximal shunting.

## 4. Discussion

This retrospective review is one of the largest contemporary series of priapism, and we have identified several novel findings. Our primary objective was to identify pertinent comorbidities. We found a high prevalence of mental illness in patients presenting with priapism (56%), with substance abuse as the most common mental health disorder. The secondary objective of the study was to assess outcomes of procedures to treat ischemic priapism. In the current study, poor followup was common, with only 44% of patients seen in clinic after the resolution of priapism. In general, return of erectile function was quite limited. 

### 4.1. Priapism Comorbidity: Mental Illness and Psychopharmaceuticals

The contribution that mental health plays in relationship to erectile physiology and priapism is largely unknown. Prior authors have reported the association of both substance abuse and mental disorders (e.g., schizophrenia) to priapism [3–5, 16]. The molecular mechanism by which certain psychopharmaceuticals and substances of abuse cause priapism is believed to be through blockade of alpha 1 adrenoceptors [5, 6]. Cocaine and methamphetamine have known affinity for adrenoreceptors. One hypothesis is that persons with substance abuse disorders and other mental illnesses may experience autonomic dysregulation [7–10] which may lead to subsequent changes in alpha receptor activity/responsivity. The sympathetic nervous system, which mediates alpha receptor activity, is primarily responsible for the detumescence response in the erectile reflex, and this may be where the dysautonomic regulation occurs with priapism. Perhaps mental illnesses and/or the psychopharmaceuticals commonly used to treat these conditions alter the central nervous system's ability to appropriately regulate the erectile response, thereby predisposing patients to priapism. 

The current study corroborates these associations and identifies new issues. In our cohort, many of the patients with prescribed psychopharmaceuticals were not taking their medication at the time of the priapism event. This would suggest priapism may be a sequel of medication withdrawal in some patients, as well as being potentiated by the same pharmaceuticals in others. Unfortunately, there is no consistent and predictable way to determine which medications or doses will cause priapism [11]. 

### 4.2. Outcomes from Priapism Treatment

The most acute complication in this cohort was atrial fibrillation requiring cardioversion, following the use of phenylephrine irrigation. To our knowledge, this complication has not been previously reported in the literature, and it appears to be an alpha adrenergically mediated side effect. Urethral injuries have been previously reported from priapism shunt surgery and were also associated with multiple interventions [12]. The 2 patients with urethral injuries in this cohort both had multiple shunt procedures. 

Priapism carries a substantial risk of erectile dysfunction, with historical reports of >35% [1, 13]. Recent modifications in shunting techniques have shown promising results in patients who had ischemic periods over 24 hours, with these patients having return of erections [14]. Of the 35 patients seen in followup in the current series, 6 reported erections adequate for intercourse after treatment. The low potency rate is likely due to sampling bias, with only the patients with ED desiring to return to clinic, but it is unlikely that all of the patients lost to followup were potent. In 5 patients with a persistent surgical shunt who underwent surgical closure for treatment of erectile dysfunction, only 2 had improvement to functional erections. Surgical closure of shunts should be considered in patients with documented patent shunts and ED. However, even surgical reversal of a shunt procedure may not restore erectile function, indicating that there is substantial tissue compromise after priapism. 

Our findings corroborate previously published work reporting that neurogenic priapism is self-limited and is not associated with complications. Our results do differ from an earlier study in that the majority of patients in our series are from subjects who have a cerebral lesion, not a spinal cord injury [15]. Hinman's seminal article from 1914 described priapism from both cerebral and spinal cord lesions [13]. One of the main differences between Hinman's series and ours is that the former included many infectious etiologies that are not as prevalent today. Our findings corroborate previous findings that, in patients who have had an acute central nervous system injury, the resultant priapism can be managed with observation alone. 

With our data culled from a single institution, there are differences in our cohort compared to other series. Most notably, our series of men with sickle cell disease is distinctly less common compared to others [14, 16–18].

## 5. Conclusion

We describe the characteristics and outcomes of a large group of patients with priapism. Our experience at a tertiary care center indicates that mental illness, including substance abuse disorders, is a highly prevalent comorbid condition in men who experience priapism. Consistent with previous reports, erectile dysfunction is the most common complication from priapism and its treatment, occurring in the majority of men.

## Figures and Tables

**Table 1:** Characteristics of priapism.

	*N*	Mean age (years)	Mean duration of priapism (hours)
Low flow	77	41	58
Neurogenic^∗^	15	35	6
High flow	2	19	252

^∗^ The neurogenic priapism patients are included in the total for “low-flow,” but are separated out for clarity.

**Table 2:** Comorbid conditions in patients with low-flow priapism^∗^.

	(*N*)
Mental illness	
Substance abuse	29
Bipolar	10
Schizophrenia/schizoaffective	5
Depression	4
Other	3
Neurological injury	
Head injury only	8
Spinal cord injury only	4
Head and spinal injury	3
Hypertension	8
HIV	4
Sickle cell anemia/trait	3
Malignancy, active	2
Hyperlipidemia	2

^∗^ Some patients with multiple diagnoses.

**Table 3:** Priapism treatments and outcomes.

Treatment	(*N*)
Observation/no treatment (incl. neurogenic)	20
Embolization	1
Irrigation only	22
Irrigation followed by shunt	36
Number of separate shunt procedures per patient	
(1)	18
(2)	15
(3)	2
(4)	1

Immediate complications	
procedure	Complication and treatment (*n*)

Irrigation and injection	Atrial fibrillation → cardioversion (1)
Distal shunt	Wound infection → antibiotics (1)
Proximal shunt	Urethral injury → prolonged catheter (2)
	Perineal hematoma → evacuation (1)
	Perineal wound infection → wound care (1)

Long-term outcomes	

Number with follow-up	35
Median follow-up (months)	3, range 0.25–60
Chronic pain	5
Further surgery	
Prosthesis placement	3
Shunt closure	5
Intermittent priapism	1
Erections inadequate for intercourse	29

## References

[1] Montague DK, Jarow J, Broderick GA (2003). American urological association guideline on the management of priapism. *Journal of Urology*.

[2] Dindo D, Demartines N, Clavien PA (2004). Classification of surgical complications: a new proposal with evaluation in a cohort of 6336 patients and results of a survey. *Annals of Surgery*.

[3] Kulmala RV, Lehtonen TA, Tammela TL (1995). Priapism, its incidence and seasonal distribution in Finland. *Scandinavian Journal of Urology and Nephrology*.

[4] Fiorelli RL, Manfrey SJ, Belkoff LH, Finkelstein LH (1990). Priapism associated with intranasal cocaine abuse. *Journal of Urology*.

[5] Munarriz R, Hwang J, Goldstein I, Traish AM, Kim NN (2003). Cocaine and ephedrine-induced priapism: case reports and investigation of potential adrenergic mechanisms. *Urology*.

[6] Andersohn F, Schmedt N, Weinmann S, Willich SN, Garbe E (2010). Priapism associated with antipsychotics: role of *α*1 adrenoceptor affinity. *Journal of Clinical Psychopharmacology*.

[7] Bär KJ, Boettger MK, Schulz S (2008). Reduced cardio-respiratory coupling in acute alcohol withdrawal. *Drug and Alcohol Dependence*.

[8] Vongpatanasin W, Mansour Y, Chavoshan B, Arbique D, Victor RG (1999). Cocaine stimulates the human cardiovascular system via a central mechanism of action. *Circulation*.

[9] Henry BL, Minassian A, Paulus MP, Geyer MA, Perry W (2010). Heart rate variability in bipolar mania and schizophrenia. *Journal of Psychiatric Research*.

[10] Carney RM, Blumenthal JA, Stein PK (2001). Depression, heart rate variability, and acute myocardial infarction. *Circulation*.

[11] Sood S, James W, Bailon MJ (2008). Priapism associated with atypical antipsychotic medications: a review. *International Clinical Psychopharmacology*.

[12] De Stefani S, Savoca G, Ciampalini S, Stener S, Gattuccio I, Belgrano E (2001). Urethrocutaneous fistula as a severe complication of treatment for priapism. *British Journal of Urology International*.

[13] Hinman F (1914). Priapism: report of cases and a clinical study of the literature with reference to its pathogenesis and surgical treatment. *Annals of Surgery*.

[14] Brant WO, Garcia MM, Bella AJ, Chi T, Lue TF (2009). T-shaped shunt and intracavernous tunneling for prolonged ischemic priapism. *Journal of Urology*.

[15] Gordon SA, Stage KH, Tansey KE, Lotan Y (2005). Conservative management of priapism in acute spinal cord injury. *Urology*.

[16] Kulmala R, Lehtonen T, Nieminen P, Tammela T (1995). Aetiology of priapism in 207 patients. *European Urology*.

[17] El-Bahnasawy MS, Dawood A, Farouk A (2002). Low-flow priapism: risk factors for erectile dysfunction. *British Journal of Urology International*.

[18] Earle CM, Stuckey BG, Ching HL, Wisniewski ZS (2003). The incidence and management of priapism in Western Australia: a 16 year audit. *International Journal of Impotence Research*.

